# Supplementary material for: Visual cortical area contributions to the transient, multifocal and steady-state VEP: A forward model-informed analysis
Source: Imaging Neurosci (Camb). 2024 Apr 26;2:imag-2-00152. doi: 10.1162/imag_a_00152 (PMC12261954; doi:10.1162/imag_a_00152)
Supplement: Supplementary Material [file imag_a_00152-supp.pdf]

SUPPLEMENTARY MATERIAL

Table S1 Latencies of positive (P) and negative (N) peaks of β-coefficients in the transient VEP for single-area models

	Polarity (P/N)	Polarity (P/N)	Polarity (P/N)	Polarity (P/N)
	**Latency (ms)**	**Latency (ms)**	**Latency (ms)**	**Latency (ms)**
**V1**	P	N	P	P
	75	*145*	*190*	*290*
**V2**	P	N	N	-
	105	*170*	*230*	-
**V3**	N	P	N	N
	70	*130*	*190*	*300*

Table S2 Latencies of peaks and troughs of β-coefficients in the transient VEP for the full model (V1-3+MT)

	Polarity (P/N)	Polarity (P/N)	Polarity (P/N)	Polarity (P/N)
	**Latency (ms)**	**Latency (ms)**	**Latency (ms)**	**Latency (ms)**
**V1**	P	N	P	P
	75	*145*	*190*	*290*
**V2**	N	P	N	N
	60	*105*	*170*	-
**V3**	N	P	N	N
	70	*130*	*215*	*305*

Table S3 Latencies of peaks and troughs of β-coefficients in the multifocal VEP for single-area models

	Polarity (P/N)	Polarity (P/N)	Polarity (P/N)

	Latency (ms)	Latency (ms)	Latency (ms)
**V1**	P	N	P
	70	*140*	*260*
**V2**	P	N	P
	85	*135*	*240*
**V3**	N	N	N
	70	*150*	*300*

Table S4 Latencies of peaks and troughs of β-coefficients in the multifocal VEP for the full model (V1-3+MT)

	Polarity (P/N)	Polarity (P/N)	Polarity (P/N)
	**Latency (ms)**	**Latency (ms)**	**Latency (ms)**
**V1**	P	N	P
	70	*135*	*255*
**V2**	N	N	-
	70	*135*	-
**V3**	N	N	-
	65	*155*	-

Table S5 Latencies of positive (P) and negative (N) peaks of β-coefficients in the transient VEP for single-area models, including MT

	Polarity (P/N)	Polarity (P/N)	Polarity (P/N)	Polarity (P/N)
	**Latency (ms)**	**Latency (ms)**	**Latency (ms)**	**Latency (ms)**
**V1**	P	N	P	P

	75	*145*	*190*	*290*
**V2**	P	N	N	-
	105	*170*	*230*	-
**V3**	N	P	N	N
	70	*130*	*190*	*300*
**MT**	N	P	P	N
	80	*150*	*225*	*280*

Table S6 Latencies of peaks and troughs of β-coefficients in the transient VEP for the full model (V1-3+MT), including MT

	**Polarity (P/N)**	**Polarity (P/N)**	**Polarity (P/N)**	**Polarity (P/N)**
	**Latency (ms)**	**Latency (ms)**	**Latency (ms)**	**Latency (ms)**
**V1**	P	N	P	P
	75	*145*	*190*	*290*
**V2**	N	P	N	N
	65	*110*	*190*	*290*
**V3**	N	P	N	N
	70	*130*	*215*	*305*
**MT**	N	P	P	N
	80	*145*	*225*	*280*

Table S7 Latencies of peaks and troughs of β-coefficients in the multifocal VEP for single-area models, including MT

	**Polarity (P/N)**	**Polarity (P/N)**	**Polarity (P/N)**
	**Latency (ms)**	**Latency (ms)**	**Latency (ms)**

**V1**	P	N	P
	70	*140*	*260*
**V2**	P	N	P
	85	*135*	*240*
**V3**	N	N	N
	70	*150*	*300*
**MT**	N	P	N
	75	*135*	*250*

Table S8 Latencies of peaks and troughs of β-coefficients in the multifocal VEP for the full model (V1-3+MT), including MT

	**Polarity (P/N)**	**Polarity (P/N)**	**Polarity (P/N)**
	**Latency (ms)**	**Latency (ms)**	**Latency (ms)**
**V1**	P	N	P
	70	*135*	*255*
**V2**	N	N	-
	70	*140*	-
**V3**	N	N	-
	65	*155*	-
**MT**	N	P	N
	75	*130*	*255*

RESULTS WITH MT INCLUDED

The models in the main text only considered visual areas V1, V2 and V3 because these are the areas implicated in the debate surrounding C1 generation (

Ales et al., 2013; Kelly, Schroeder, et al., 2013; Kelly, Vanegas, et al., 2013) and because these are the only areas in the Benson-2014 retinotopy atlas known to reverse in polarity across the horizontal meridian. However, in one previous SSVEP source analysis study, a prominent contribution from MT was identified in addition to V1 (Di Russo et al., 2007). Therefore, to follow up both the static and time-resolved models, we fit a model including area MT in addition to V1, V2 and V3, though the results of it should be treated with caution because MT retinotopy carries considerably more uncertainty than V1-3 since the MT complex comprises a number of smaller sub-areas with larger receptive field sizes (Amano et al., 2009; Kolster et al., 2010; Pitzalis et al., 2010). This model explained 57% of variance in the transient C1 (increase of 1% compared with the V1-3 model) with an MT weight of -0.12, 58% (increase of 2%) in the multifocal C1 with an MT weight of -0.14, 55% (no change) in the 18.75 Hz SSVEP with an MT weight of -0.06, and 38% (increase of 4%) in the 7.5 Hz SSVEP with an MT weight of -0.24. This led to a drop in BIC compared to the V1-3 model in all cases, but the drop was largest for the 7.5 Hz SSVEP (Figure 7B, Table 1). Meanwhile, models with MT alone explained 11% of variance in the transient C1, 9% in the multifocal C1, 2% in the 18.75 Hz SSVEP and 22% in the 7.5 Hz SSVEP.

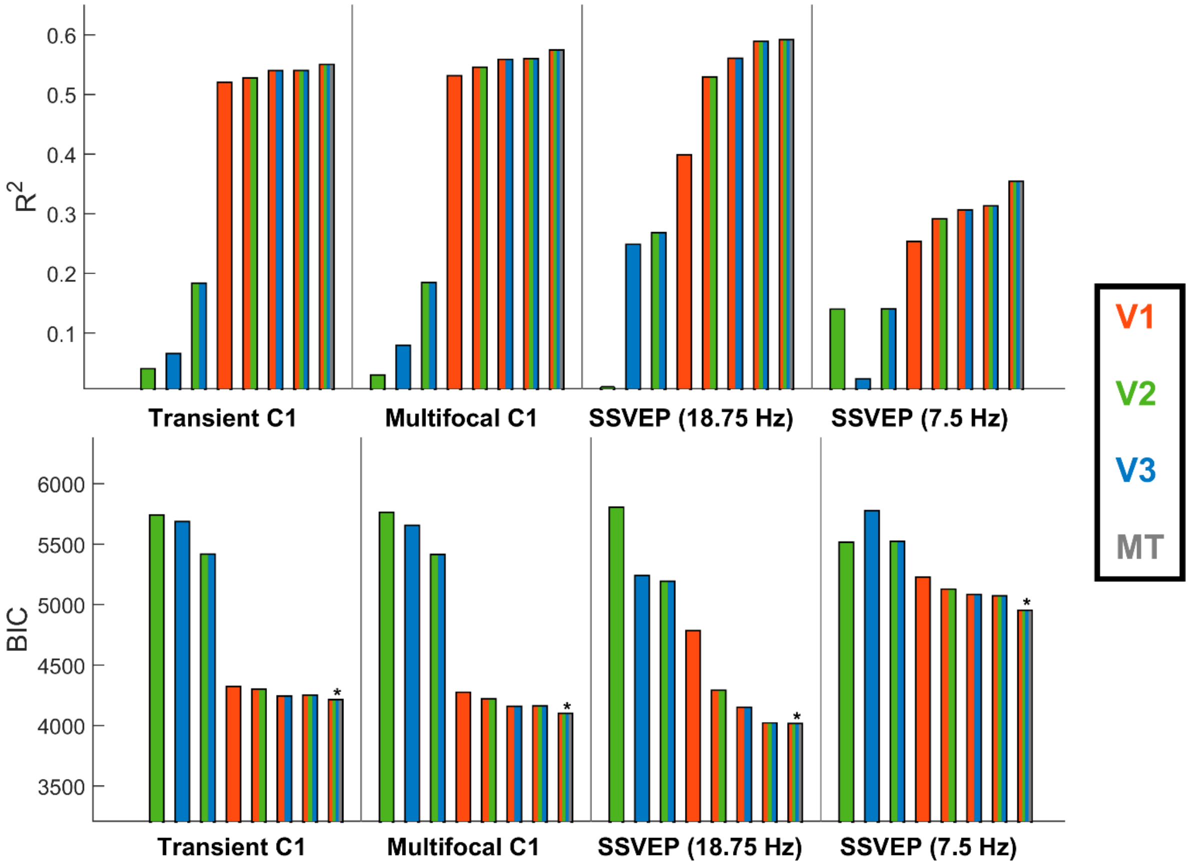

Figure S1: Regression models fitting each empirical VEP signal based on predicted topographies for V1, V2, V3 and MT, showing R^2 (A) and BIC values (B). Each panel corresponds to a signal and each bar to a model, with the set of colours within each bar denoting the visual areas included. The model with the lowest BIC for each signal is marked with an asterisk.

Table S5: Beta coefficients, R^2, and BIC values of linear regression models predicting empirical VEP signals from MRI-predicted topographies for V1-3 and MT.

		V1	V2	V3	V1&V2	V1&V3	V2&V3	V1-3	V1-3 +MT
**Transient C1**	β_{V1}	0.72	-	-	0.76	0.7	-	0.7	0.67
	β_{V2}	-	0.2	-	-0.09	-	0.38	-0.01	-0.06
	β_{V3}	-	-	-0.25	-	-0.14	-0.42	-0.14	-0.15
	β_{MT}	-	-	-	-	-	-	-	-0.12
	R^2	0.52	0.04	0.06	0.53	0.54	0.18	0.54	0.55
	**BIC**	4286	5760	5663	4241	4175	5409	4180	4142
**Multifocal C1**	β_{V1}	0.73	-	-	0.78	0.7	-	0.72	0.68
	β_{V2}	-	0.17	-	-0.13	-	0.36	-0.04	-0.1
	β_{V3}	-	-	-0.28	-	-0.17	-0.44	-0.15	-0.16
	β_{MT}	-	-	-	-	-	-	-	-0.15
	R^2	0.53	0.03	0.08	0.54	0.56	0.18	0.56	0.57
	**BIC**	4269	5757	5650	4214	4153	5409	4155	4095
**SSVEP (18.75 Hz)**	β_{V1}	0.63	-	-	0.78	0.57	-	0.67	0.65
	β_{V2}	-	-0.09	-	-0.39	-	0.16	-0.22	-0.24
	β_{V3}	-	-	-0.5	-	-0.41	-0.57	-0.3	-0.3
	β_{MT}	-	-	-	-	-	-	-	-0.06
	R^2	0.4	0.01	0.25	0.53	0.56	0.27	0.59	0.59
	**BIC**	4778	5799	5234	4286	4145	5187	4017	4011
**SSVEP**	β_{V1}	0.5	-	-	0.42	0.54	-	0.49	0.42

**(7.5 Hz)**	β_{V2}	-	0.37	-	0.21	-	.38	0.11	0.02
	β_{V3}	-	-	0.15	-	0.23	-0.02	0.18	0.15
	β_{MT}	-	-	-	-	-	-	-	-0.25
	R^2	0.25	0.14	0.02	0.29	0.31	0.14	0.31	0.35
	**BIC**	5222	5511	5771	5122	5078	5517	5066	4948

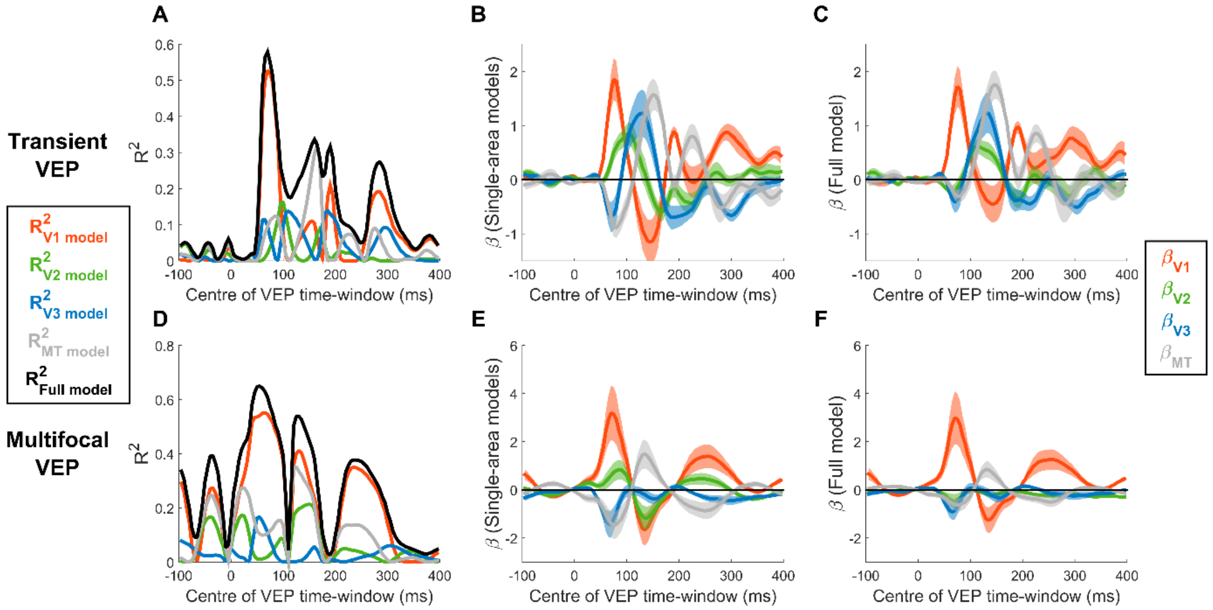

Figure S2: Model fits of the transient VEP (**A-C**) and the multifocal VEP (**D-F**) as a function of the centre-point of the 10-ms signal measurement window. **A&D)** R^2 values across time for single-area models (V1, V2, V3 and MT) and the full model including all four visual areas. **B&E)** β-coefficients ±1 bootstrap standard error for each of these four visual areas in the single-area models. **C&F)** β-coefficients ±1 standard error for each of these four visual areas in the full model.

Table 2: Pearson's correlation coefficients among the MRI-predicted topographies of V1, V2, V3 and MT.

	**V2**	**V3**	**MT**
**V1**	0.38	-0.16	-0.41
**V2**	-	0.44	-0.51
**V3**	-	-	-0.21

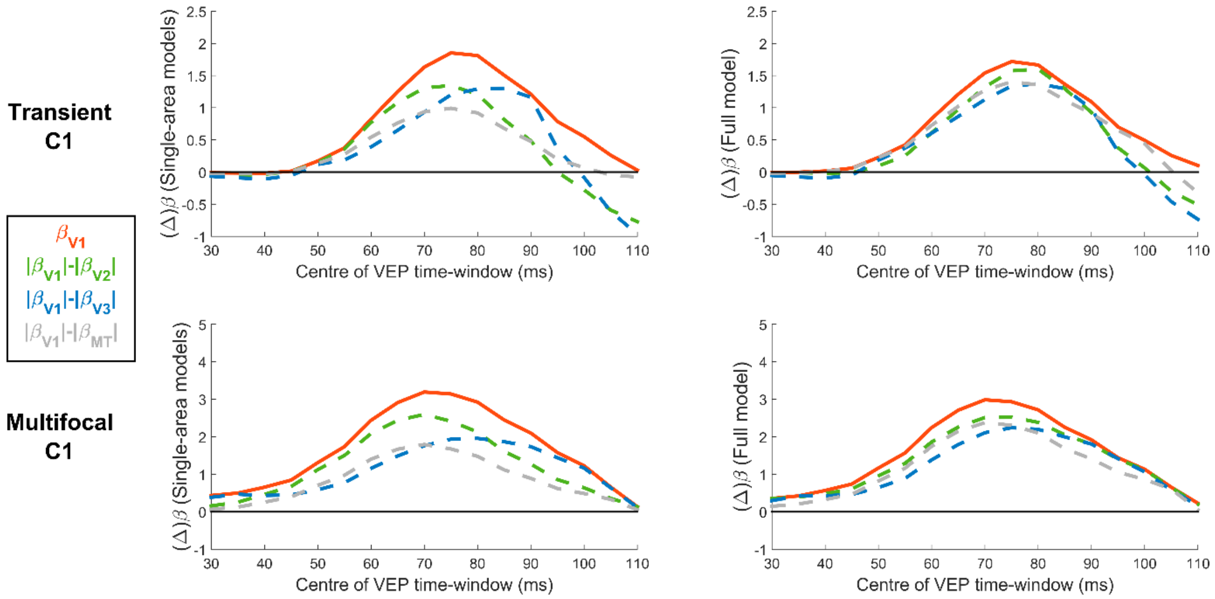

Figure S3: Difference in absolute β-coefficients of V1 relative to V2, V3 and MT during the C1 time frame for the transient and multifocal C1 and for single-area and full models. These β-differences are plotted alongside the β-coefficients of V1 itself to aid in the comparison of peak times, demonstrating that V1 β-coefficients are maximally different from those of the other three areas around the time of the C1 peak.

As with the static model, a follow-up model was carried out with MT additionally included for the time-resolved model too in light of the previous implication of MT in the SSVEP (Di Russo et al., 2007). Baseline phases and time lags were taken from the original model and fixed, allowing only the time lag of MT and the magnitudes of all four visual areas to vary. This was done for reasons of computation time given the combinatorial nature in which time lags were assigned to visual areas across regression models. Overall R^2 increased from 0.39 to 0.41. This increase was achieved primarily through an improved fit of the 7.5 Hz SSVEP, for which R^2 increased from 0.39 to 0.41, while R^2 for the 18.75 Hz SSVEP remained at 0.47. MT lagged V1 by 8 ms and its weight was -0.09 for the 18.75 Hz SSVEP (compared to 0.84 for V1) and -0.26 for the 7.5 Hz SSVEP (compared to 0.68 for V1).

Ales, J. M., Yates, J. L., & Norcia, A. M. (2013). On determining the intracranial sources of visual evoked potentials from scalp topography: A reply to Kelly et al. (this issue). *NeuroImage*, *64*, 703–711. https://doi.org/10.1016/j.neuroimage.2012.09.009

Amano, K., Wandell, B. A., & Dumoulin, S. O. (2009). Visual Field Maps, Population Receptive Field Sizes, and Visual Field Coverage in the Human MT+ Complex. *Journal of Neurophysiology*, *102*(5), 2704–2718. https://doi.org/10.1152/jn.00102.2009

Di Russo, F., Pitzalis, S., Aprile, T., Spitoni, G., Patria, F., Stella, A., Spinelli, D., & Hillyard, S. A. (2007). Spatiotemporal analysis of the cortical sources of the steady-state visual evoked potential. *Human Brain Mapping*, *28*(4), 323–334. https://doi.org/10.1002/hbm.20276

Kelly, S. P., Schroeder, C. E., & Lalor, E. C. (2013). What does polarity inversion of extrastriate activity tell us about striate contributions to the early VEP? A comment on Ales et al. (2010). *NeuroImage*, *76*, 442–445. https://doi.org/10.1016/j.neuroimage.2012.03.081

Kelly, S. P., Vanegas, M. I., Schroeder, C. E., & Lalor, E. C. (2013). The cruciform model of striate generation of the early VEP, re-illustrated, not revoked: A reply to Ales et al. (2013). *NeuroImage*, *82*, 154–159. https://doi.org/10.1016/j.neuroimage.2013.05.112

Kolster, H., Peeters, R., & Orban, G. A. (2010). The Retinotopic Organization of the Human Middle Temporal Area MT/V5 and Its Cortical Neighbors. *Journal of Neuroscience*, *30*(29), 9801–9820. https://doi.org/10.1523/JNEUROSCI.2069-10.2010

Pitzalis, S., Sereno, M. I., Committeri, G., Fattori, P., Galati, G., Patria, F., & Galletti, C. (2010). Human V6: The Medial Motion Area. *Cerebral Cortex*, *20*(2), 411–424. https://doi.org/10.1093/cercor/bhp112
